# A Rare B-Myeloid Conversion of Follicular Lymphoma into Clonally Related Acute Myeloid Leukemia: A Case Report

**DOI:** 10.3390/life13030729

**Published:** 2023-03-08

**Authors:** Xiyue Yan, Juan Liu, Yu Ben, Weicheng Zheng, Pan Hu, Yaping Zhang, Wenyu Shi

**Affiliations:** 1 Department of Nursing, Nantong Health College of Jiangsu Province, Nantong 226001, China; 2 Department of ICU, Nanjing Pukou District Central Hospital, Nanjing 211800, China; 3 Department of Hematology, Affiliated Hospital of Nantong University, Nantong 226001, China; 4 Department of Oncology, Affiliated Hospital of Nantong University, Nantong 226001, China

**Keywords:** transformation, follicular lymphoma, myeloid leukemia, PET/CT, BCL-2

## Abstract

Follicular lymphoma (FL) is a highly prevalent indolent lymphoma, and the risk of histological transformation is approximately 2–3% per year. Transformation of FL generally occurs in the same lineage (B cell lineage). Another rare form of disease progression is the transformation of neoplastic B-cells to another cell lineage such as acute myeloid leukemia (AML). The low incidence of B-myeloid transformation associated with poor prognosis hinders the establishment of model systems to identify molecular mechanisms. A 64-year-old woman was diagnosed with FL and achieved a satisfactory response after six cycles of R-CHOP (rituximab, cyclophosphamide, doxorubicin, vincristine, and prednisone). Approximately one month after treatment terminated, the disease progressed to AML with an increased white blood cell count and abnormal coagulation. Interestingly, nucleotide sequence analysis of the genomic region encoding the immunoglobulin heavy-chain variable domain showed the possibility of homologous transformation from lymphoma to leukemia cells. Although the patient experienced transient improvement after undergoing treatment with one cycle of idarubicin and cytarabine combined with etoposide, she relapsed and died 8 days after venetoclax salvage therapy. Patient with B-myeloid transformation was associated with an aggressive clinical course and poor prognosis. Conventional strategies for treating histologically transformed AML were ineffective. However, treatment with a Bcl-2 inhibitor could serve as an option. Here we review the literature relevant to this rare histological transformation of FL.

## 1. Background

Follicular lymphoma (FL) is generally an indolent B-cell lymphoproliferative disorder that transforms follicular center B cells. FL accounts for 5% of all hematologic neoplasms. FL is the second most frequent non-Hodgkin lymphoma (NHL) subtype, which accounts for approximately 10–20% of lymphomas. FL occurs slightly more commonly in males than in females (sex ratio 1.2:1). It is more common in the elderly, with a median age at diagnosis ranging from 60 to 65 years, and the incidence gradually increases from 35 years to a peak at 70 years. [1] It is characterized by a variety of clinical behaviors and the risk of recurrence and histological changes. The annual risk of histological transformation is approximately 2–3%. Transformation is associated with rapid progression of lymphadenopathy, extra-nodal disease (besides the marrow), B symptoms, hypercalcemia, elevated serum LDH, high FLIPI score, and non-response to initial therapy. It is well known that FL histologically transforms into a more aggressive form of NHL, and the most typical type is diffuse large B-cell lymphoma [2]. Another rare form of disease progression is the transformation of neoplastic B-cells to another cell lineage that gives rise to histiocytic sarcoma or acute myeloid leukemia (AML) [2,3]. Although we have already known the transformation from lymphoma to myeloid neoplasm and this form of transformation heralds a very poor prognosis, the low incidence of B-myeloid transformation associated with poor prognosis hinders the establishment of model systems to identify the molecular mechanisms.

Acute myeloid leukemia (AML) is a hematologic malignancy that is characterized by clonal proliferation of poorly differentiated cells of blood origin. These cells undergo genetic alterations, including repetitive deletions, amplifications, point mutations, and rearrangements [4]. In United States’ adults, the incidence of AML was higher than that of the other three leukemia subtypes (acute lymphoblastic leukemia (ALL), chronic myelogenous leukemia (CML), and B-cell chronic lymphocytic leukemia (CLL)) until 2017, when it was surpassed by CLL. Among all leukemia subtypes, AML accounts for the highest proportion of leukemia deaths (62%) [5]. AML is a heterogeneous disease, defined by extensive genomic changes and molecular mutations that impact clinical outcomes and provide potential targets for drug development. Conventional strategies for treating histologically transformed AML may be ineffective, while Bcl-2 inhibitors may serve as an alternative option for this situation. BCL-2 is a member of the BCL-2 family of anti-apoptotic and pro-apoptotic proteins and protects cells from apoptosis. BCL-2 expression in AML is associated with reduced sensitivity to cytotoxic chemotherapy and higher recurrence rates. Venetoclax is a selective BCL-2 inhibitor that is orally bioavailable. It promotes intrinsic apoptotic pathway activation through BCL-2-mediated separation of the BH3 proteins Bim and BID and the effector proteins Bax and Bak, resulting in outer mitochondrial membrane permeability [6].

Here we report a rare case of myeloid transformation of FL. The patient achieved six cycles of R-CHOP (rituxan, cyclophosphamide, vinorelbine, doxorubicin liposome, and prednisone acetate), and her condition was alleviated, but the disease eventually progressed into AML. The patient was therefore administered salvage therapy comprising dose-escalation venetoclax combined with decitabine, homo-harringtonine, and low doses of cytarabine. Unfortunately, her condition rapidly deteriorated, and she died due to multiple organ failures on day 8 after venetoclax-combined chemotherapy.

## 2. Case Presentation

**Figure 1:**
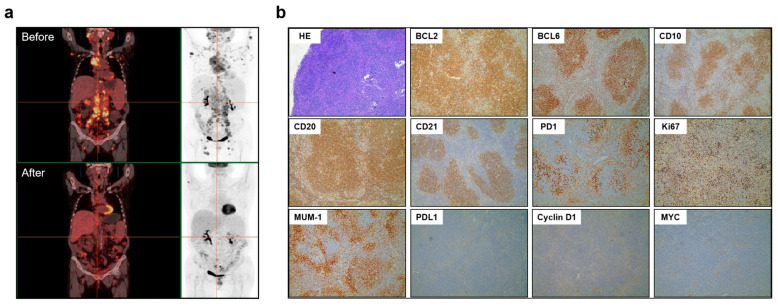
Positron emission tomography/computed tomography scans revealed a satisfactory response after six cycles of R-CHOP treatment (**a**). Results of immunohistochemical staining of follicular lymphoma tissue samples (**b**). Hematoxylin and eosin staining, ×100 magnification. Neoplastic cells were positive for Bcl-2, Bcl-6, CD10, CD20, CD21, PD1, Ki67(20%), MUM-1, and negative for PDL1, Cyclin D1 and MYC. Original magnification, ×100. (Hematoxylin and eosin staining ×400 magnification is shown in Supplementary Material Figure S2).

A 64-year-old woman was treated at our hospital for more than half a year of cervical lymphadenopathy. She lost more than 10% of her body weight in nearly 1 year and did not experience fever or night sweats during the disease. The patient had a 3-year history of diabetes, which was controlled. Clinical examination revealed multiple cervical lymph nodes. Abnormal laboratory test results showed white blood cell count, 4.8 × 10^9^/L; Hb, 103 g/L; MCV, 76.3fl; and platelet count, 86 × 10^9^/L. Serum lactate dehydrogenase and Serum β2-microglobulin levels were 244 U/L (reference range 100–235 U/L) and 5.6 mg/L (reference range 0.8–2.4 mg/L), respectively). Computed Tomography of the local hospital showed two lung nodules with mediastinal lymph node metastasis and multiple abdominal lymph nodes. Positron emission tomography/computed tomography (PET/CT) detected heterogeneously sized enlarged lymph nodes that were diffusely distributed above and below the diaphragm, with multiple sites of bone destruction (Figure 1a).

Multiple celiac lymph nodes (approximately 0.8–3.2 cm in diameter) showed the highest standard uptake value (18.2) (Figure 1a). The total metabolic tumor volume (TMTV) and total lesion glycolysis (TLG) of the four larger lesions were 240.45 cm^3^ and 990.94 g, respectively. A biopsy of the right mandibular lymph node revealed follicular hyperplasia of lymphoid tissue. Immunocytochemistry detected Ki67 (20%), CD10, Bcl-2, Bcl-6, SOX11, CD21, CD20, CD79a, MUM-1 (partially), and PD1 (partially) but not CD5, CD3, PDL1, MYC, and cyclin D1 (Figure 1b). Fluorescence in situ hybridization (FISH) analysis of bone marrow revealed a rearrangement involving the BCL2 and IGH genes (BCL2/IGH) in 10% of cells.

The patient was accordingly diagnosed with FL (low-grade) stage IV, group B (Follicular Lymphoma International Prognostic Index (FLIPI), 5 points, high risk). At this point, the patient’s performance status was ECOG1. After four cycles of R-CHOP (rituximab, cyclophosphamide, doxorubicin, vincristine, and prednisone), interim evaluation using enhanced CT showed partial remission. The patient achieved a satisfactory response after six cycles of R-CHOP (evaluated using PET/CT) (Figure 1a), except for two suspicious small lesions that presented as a new 0.8-cm nodule (SUVmax = 10.4) in the deltoid muscle of the right shoulder and as a new 1.0-cm nodule (SUVmax = 6.3) in the right ilium (Supplementary Material Figure S1). Abnormal laboratory test results showed white blood cell count, 3.4 × 10^9^/L; Hb, 113 g/L; MCV, 79.1fl; and platelet count, 134 × 10^9^/L.

**Figure 2:**
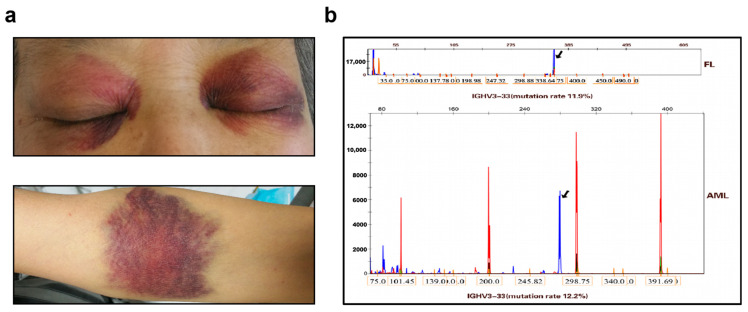
Ecchymosis of the skin (**a**). The igH clone peak detected from the peripheral blood sample of AML was consistent with that in FL tissue (**b**).

Approximately 1 month after the above treatment, the disease progressed to AML, with scattered ecchymoses (Figure 2a), accompanied by abnormal laboratory test results (white blood cell count, 41.2 × 10^9^/L; monocyte count, 13.23 × 10^9^/L (32.1%); Hb, 123g/L; MCV, 86fl; and platelet count, 43 × 10^9^/L). By now, the patient’s performance status was ECOG3. Serum lactate dehydrogenase and D-dimer levels were extremely high (3575 U/L (reference range 100–235 U/L) and 3.55 mg/L FEU (reference range 0–0.85 mg/L FEU), respectively). A bone marrow smear showed significant nucleated cell proliferation, accounting for 72% of blasts. Peroxidase staining results were negative. Flow cytometric analysis of the marrow showed 66.13% promyelocytes with positive markers of CD15, CD33, CD64, HLA-DR, CD56, and CD11b (partial positive), and negative makers of CD34, CD117, CD13, CD5, CD7, CD10, CD19, CD20 and CD22, which was consistent with the immunophenotype of AML (AML-M5 or Acute monoblastic and monocytic leukemia based on WHO classification) (Supplementary Material Figures S3 and S4). FISH analysis of bone marrow detected BCL-2/IGH (35%) rearrangement and C-MYC (40%) rearrangement, but not BCL-6, MLL, P53, or BCR/ABL.

We next analyzed a FL tissue sample and an AML peripheral blood sample to detect the immunoglobulin heavy-chain variable region (IGHV) gene rearrangement. The FL sample showed the highest homology with IGHV3-33, with 88.1% identical nucleotide sequences. Sequencing of the transformed myeloid leukemia sample showed the highest degree of homology at IGHV3-33, with 87.8% identical nucleotide sequences (Figure 2b). Notably, after six cycles of R-CHOP, lymphocyte subsets represented 0.07% (reference range 3–15%) of B lymphocytes.

A similar result of the IGHV test indicated the possibility of homologous transformation of FL to AML. Second-generation gene sequencing revealed mutations in KMT2D, EP300, ARID1A, CREBBP, NRAS, PDGFRA, and NCOR2. After one cycle of idarubicin and cytarabine combined with etoposide, ecchymoses were partially absorbed, the white blood cell count returned to normal, and lactate dehydrogenase levels decreased to 486 U/L. After transient improvement, the disease recurred, with a persistently high fever and an extremely low platelet count. A bone marrow smear showed significant nucleated cell proliferation, accounting for 82.5% of blasts. Flow cytometric analysis of the marrow showed 66.36% promyelocytes with positive markers of CD33, CD15, HLA-DR, CD56, CD64, and CD13 (partial positive), and negative makers of CD5, CD7, CD10, CD19, CD11b, CD34, CD117, and CD2, which was consistent with the immunophenotype of AML (AML-M5 or Acute monoblastic and monocytic leukemia based on WHO classification). The patient was therefore administered salvage therapy comprising dose-escalation venetoclax combined with decitabine, homo-harringtonine, and low doses of cytarabine. However, her condition rapidly deteriorated, accompanied by abnormal laboratory tests results (white blood cell count, 59.9 × 10^9^/L; monocyte count, 23.36 × 10^9^/L (39%); Hb, 58g/L and platelet count, 69 × 10^9^/L). Serum lactate dehydrogenase and D-dimer levels were high (1706 U/L (reference range 100–235 U/L) and 1.36 mg/L FEU (reference range 0–0.85 mg/L FEU), respectively). The Fibrinogen level was extremely low (0.41 g/L (reference range 2–4 g/L)). Unfortunately, she died due to multiple organ failures on day 8 after venetoclax-combined chemotherapy.

## 3. Discussion and Conclusions

Generally speaking, the transformation of FL occurs in the same lineage (B cell lineage). Another rare form of transformation is from B-cell neoplasm to another cell lineage, such as acute myeloid leukemia [3]. The low incidence of B-myeloid transformation associated with poor prognosis hinders the establishment of model systems to identify molecular mechanisms. A 64-year-old woman was diagnosed with FL and achieved a satisfactory response after six cycles of R-CHOP (rituximab, cyclophosphamide, doxorubicin, vincristine, and prednisone). Approximately one month after treatment terminated, the disease progressed to AML, with an increased white blood cell count and abnormal coagulation. Interestingly, nucleotide sequence analysis of the genomic region encoding the immunoglobulin heavy-chain variable domain showed the possibility of homologous transformation from lymphoma to leukemia cells. Although the patient experienced transient improvement after undergoing treatment with one cycle of idarubicin and cytarabine combined with etoposide, she relapsed and died 8 days after venetoclax salvage therapy. Patient with B-myeloid transformation was associated with an aggressive clinical course and poor prognosis. Conventional strategies for treating histologically transformed AML were ineffective. However, treatment with a Bcl-2 inhibitor could serve as an option.

The underlying mechanisms of transformed AML from B-cell neoplasm are still controversial because of the low incidence of FL and a shortage of suitable in vivo models. Three hypotheses explain the mechanism of myeloid transformation of B cells as follows: 1. direct transformation (one cell type transforms into another without an intermediate progenitor stage); 2. dedifferentiation (transformation into progenitor) followed by redifferentiation along the myeloid pathway; and 3. divergent evolution from common progenitor cells [7]. The second mechanism may explain the IGHV sequencing data and second-generation sequencing results described here.

Thus, a subpopulation of FL-like B cells leaves the germinal center and returns to bone marrow niches, and then dedifferentiates into pre-B cells in which Myc upregulated expression of the insulin receptor, insulin-like growth factor 1 receptor, or both activates NF-κB signaling [7,8]. Furthermore, NRAS upregulates miR-146a, which targets NUMB mRNA, which encodes a repressor of Notch signaling, to activate Notch signaling [9]. Coactivation of NF-κB and Notch signaling may induce the B cell-to-myeloid conversion [7]. BTB Domain and CNC Homolog 2 (Bach2), a suppressor of lymphoma and myeloid-specific gene expression, serves as a downstream target of NF-κB/Notch signaling, and its expression is repressed through the coactivation of these signaling pathways [7].

Downregulation of Bach2 is an early event in B-myeloid transformation [7]. The forced expression of the myeloid transcription factor, CCAAT/enhancer-binding protein alpha (C/EBPα), inhibits B cell transcription factor Pax5 and upregulates Mac-1 and other myeloid markers to promote B–myeloid reprogramming when co-expressed with the transcription factors Oct4, Sox2, Klf4, and Myc [10,11]. The mechanism of histological transformation is complex, particularly that of the trans-lineage transformation, and therefore requires further experimental validation.

Several clinical risk models have been proposed to predict the outcome of FL. The development of next-generation sequencing (NGS) technology allows for the integration of somatic gene mutations into clinical scores to build genotyping-based risk models such as the m7-FL International Prognosis Index (FLIPI). The m7-FLIPI combines performance status, FLIPI score, and seven gene mutation status to classify patients into “low risk” and “high risk”, which is related to the 5-year failure-free survival rate after first-line immunochemotherapy. The m7-FLIPI can improve the prognosis of patients with FL undergoing first-line immunochemotherapy, and is a promising model to identify the subtype with the highest risk of treatment failure [12]. The m7-FLIPI contains EP300, ARID1A, and CREBBP mutations that are also found in other diseases, including the myeloid system. CREBBP mutation is recurrent in 66% of FL, acting by inactivating its histone acetyltransferase domain or truncating the protein. One experiment demonstrated that these two types of mutations produced varying degrees of epigenomic damage: histone acetyltransferase mutations were more severe, and the clinical prognosis was poor [13]. Xie et al. [14] reported that KAT6A-CREBBP rearrangements associated with AML usually exhibited monocyte or bone marrow monocyte differentiation and often appeared in patients with a history of treatment with cytotoxic therapies. This finding may be related to the transition of disease from FL to AML, where enhanced CREBBP and EP300 function may lead to a shift in gene transcription from transcriptional activation to aberrant repression, interfering with critical gene expression programs that control the selection, exit and differentiation of normal GC.

Conventional treatment of histologically transformed AML is largely ineffective and requires new targeting drugs; a Bcl-2 inhibitor provides a good treatment option. For example, venetoclax shows the greatest potential for effectively treating FL. The CAVALLI phase 1b trial found that 83.3% of patients with FL who received venetoclax combined with R-CHOP or G-CHOP achieved an overall response, and 75% achieved a complete response [15]. Moreover, 54% of AML patients treated with venetoclax combined with low-dose cytarabine achieved a complete response or a complete response with incomplete blood count recovery and median overall survival of 10.1 months [16]. In a trial conducted using venetoclax and azacitidine or decitabine, 61% of patients with AML achieved a complete response with or without incomplete blood count recovery [17]. These findings show that targeting Bcl-2 is an effective and well-tolerated clinical strategy for treating AML. Unfortunately, venetoclax as salvage therapy fails to improve prognosis, which may be explained by relapse and the intractable condition of this patient, preventing the administration of an effective dosing regimen. Early treatment with venetoclax of patients with histologically transformed AML may therefore serve as an alternative therapy. Furthermore, complex tumor molecular pathways might also be involved. A study on AML leukemia stem cells (LSCs) found that inhibition of amino acid metabolism is the key activity of venetoclax, which in turn led to the downregulation of oxidative phosphorylation and selective targeting of LSCs. However, in patients who fail to respond to venetoclax-based treatment, an alternative, the fatty acid metabolic pathway, can be used to drive oxidative phosphorylation [18].

In summary, B-myeloid conversion of follicular lymphoma into clonally related AML is extremely rare. Coactivation of NF-κB and Notch signaling is sufficient to lead B-myeloid conversion. Patients with this transformation are associated with an aggressive clinical course and poor prognosis. The m7-FLIPI can improve the prognosis of patients with FL undergoing first-line immunochemotherapy, and is a promising model to identify the subtype with the highest risk of treatment failure. PET/CT facilitates the identification of suspicious lesions, and conventional strategies for treating histologically transformed AML may be ineffective, while Bcl-2 inhibitors may serve as an alternative option for this situation. Due to the complexity of the transformation mechanism and individual differences in metabolic properties, the therapeutic effects of Bcl-2 inhibitors vary from person to person and further clinical studies are needed.

## Data Availability

Not applicable.

## Supplementary Materials

The following supporting information can be downloaded at: https://www.mdpi.com/article/10.3390/life13030729/s1. Figure S1. A new nodule with a diameter of 0.8 cm (SUVmax = 10.4) in the deltoid muscle of right shoulder (A) and another new nodule with a diameter of 1.0 cm (SUVmax = 6.3) in the right ilium (B). Figure S2. Hematoxylin and eosin staining, ×400 magnification. This is the ×400 magnification of HE staining (×100) in Figure 1b in the main text. Figure S3. Marrow flow cytometry indicated that promyelocytes accounted for 66.13%, cell phenotype: CD5-/CD7-/CD10-/CD19-/CD11b (partial+)/HLA DR+/CD117-/CD15+/CD33+/CD34-/CD56+/CD13-/CD64+/CD20-/CD22-, in line with acute myeloid leukemia (AML-M5). Figure S4. Bone marrow smear showed nucleated cell proliferation was significantly active, blasts accounted for 72%; peroxidase staining was negative.

## Abbreviations

FL: Follicular lymphoma: NHL: Non-Hodgkin lymphoma; AML: Acute myeloid leukemia; R-CHOP: rituximab, cyclophosphamide, doxorubicin, vincristine, and prednisone; PET/CT: Positron emission tomography /computed tomography; TMTV: Total metabolic tumor volume; TLG: Total lesion glycolysis; FISH: Fluorescence in situ hybridization; IGHV: Immunoglobulin heavy-chain variable region; Bach2: BTB Domain and CNC Homolog 2; C/EBPα: CCAAT/enhancer-binding protein alpha.
