# Supplementary material for: A Rare B-Myeloid Conversion of Follicular Lymphoma into Clonally Related Acute Myeloid Leukemia: A Case Report
Source: Life (Basel). 2023 Mar 8;13(3):729. doi: 10.3390/life13030729 (PMC10055921; doi:10.3390/life13030729)
Supplement: Supplementary file 1 [file life-13-00729-s001.zip › life-2121878-supplementary.pdf]

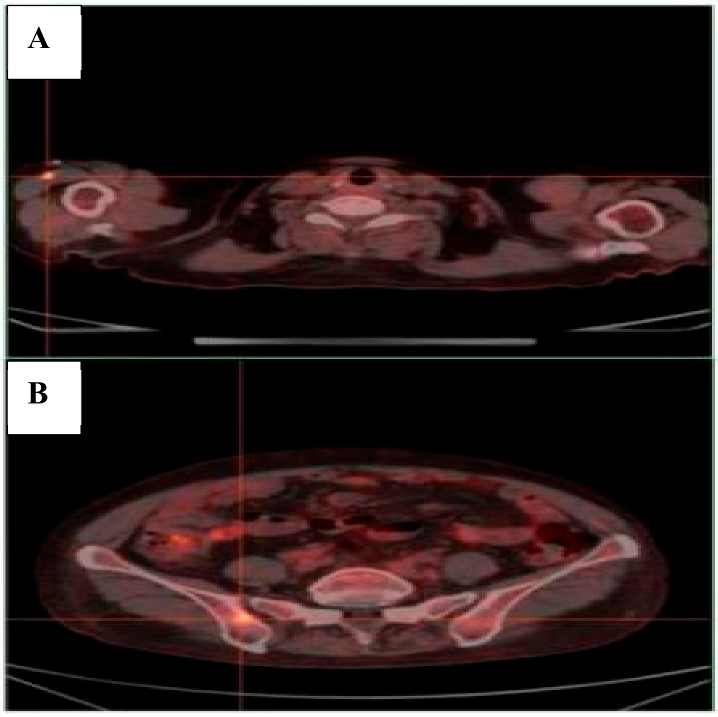

Figure S1-supplementary material. A new nodule with a diameter of 0.8 cm (SUVmax = 10.4) in the deltoid muscle of right shoulder (A) and another new nodule with a diameter of 1.0 cm (SUVmax = 6.3) in the right ilium (B).

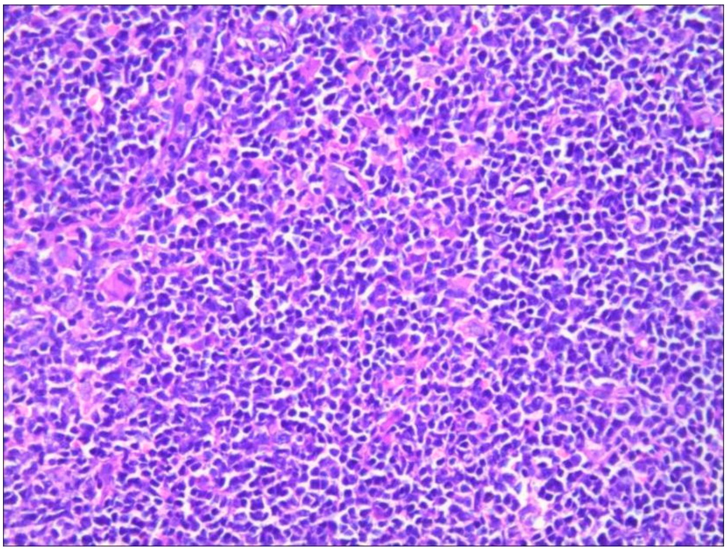

Figure S2-supplementary material. Hematoxylin and eosin staining, ×400 magnification. This is the ×400 magnification of HE staining (×100) in Figure-1b in the main text.

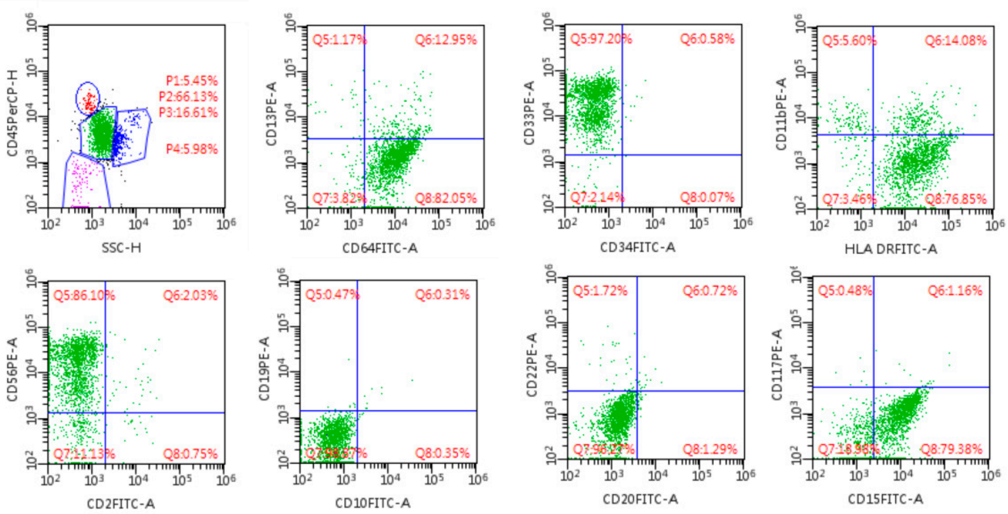

Figure S3-supplementary material. Marrow flow cytometry indicated that promyelocytes accounted for 66.13%, cell phenotype: CD5- / CD7- / CD10-/ CD19- / CD11b (partial +) / HLA DR + / CD117- / CD15 + / CD33 + / CD34-/ CD56 + / CD13- / CD64 + / CD20- / CD22-, in line with acute myeloid leukemia (AML-M5).

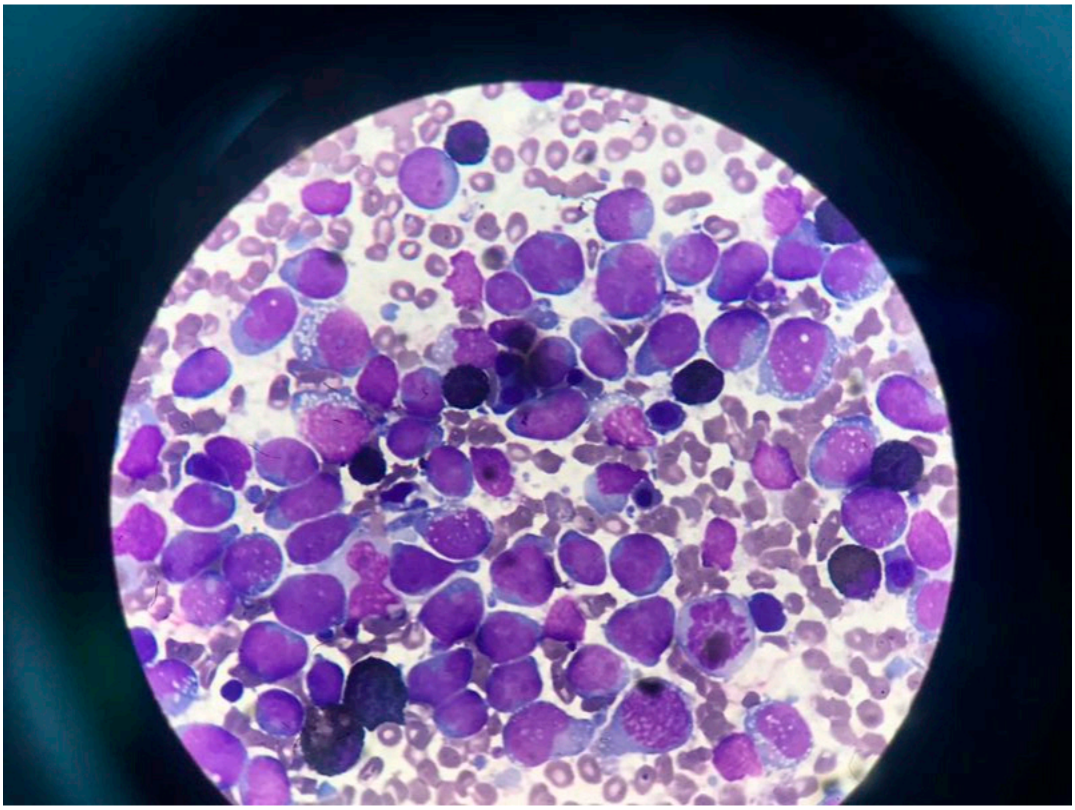

Figure S4-supplementary material. Bone marrow smear showed nucleated cell proliferation was significantly active, blasts accounted for 72%; peroxidase staining was negative.
